# Minimal extrathyroidal extension is associated with lymph node metastasis in single papillary thyroid microcarcinoma: a retrospective analysis of 814 patients

**DOI:** 10.1186/s12957-022-02629-8

**Published:** 2022-05-28

**Authors:** Ra-Yeong Song, Hee Sung Kim, Kyung Ho Kang

**Affiliations:** 1 Department of Surgery, Chung-Ang University Hospital and Chung-Ang University College of Medicine, 102 Heukseok-ro, Dongjak-gu, Seoul, 06973 Republic of Korea; 2 Department of Pathology, Chung-Ang University Hospital and Chung-Ang University College of Medicine, Seoul, Republic of Korea; 3 Department of Surgery, Ewha Womans University Medical Center, Seoul, Republic of Korea

**Keywords:** Papillary thyroid Microcarcinoma, Extrathyroidal extension, LN metastasis

## Abstract

**Background:**

Extrathyroidal extension (ETE) is considered a major prognostic factor in papillary thyroid carcinoma (PTC). Patients with gross ETE are at increased risk of recurrence and mortality. The importance of minimal ETE still remains controversial, especially in patients with papillary thyroid microcarcinoma (PTMC). The purpose of this study was to evaluate the association between ETE and lymph node (LN) metastasis in single PTMC.

**Methods:**

A retrospective analysis was performed of 1994 patients underwent thyroidectomy for PTC between 2012 and 2016 in a single institution. Patients with combined thyroid carcinoma of other types and those who underwent completion thyroidectomy were excluded. After further exclusion of PTC larger than 1 cm and multifocal tumors, 814 patients with single PTMC were included in the study.

**Results:**

72.9% patients had no ETE, 25.1% minimal ETE, and 2.1% gross ETE. ETE was associated with lymphatic invasion, perineural invasion, and vascular invasion. Patients with minimal and gross ETE were also more likely to have LN metastasis, including lateral neck metastasis, compared to those without ETE. In univariate analysis, LN metastasis was associated with male gender, conventional PTC, lymphatic invasion, perineural invasion, and ETE. In multivariate analysis, male gender (OR = 1.987; 95% CI 1.369–2.884), lymphatic invasion (OR = 4.389; 95% CI 1.522–12.658), perineural invasion (OR = 6.545; 95% CI 1.262–33.948), and minimal ETE (OR = 1.852; 95% CI 1.298–2.643) were found to be independent risk factors of LN metastasis.

**Conclusions:**

Minimal ETE is associated with LN metastasis in single PTMC, compared to no ETE. Minimal ETE should be considered in the management of patients with single PTMC, whether surgical or during active surveillance.

## Background

Extrathyroidal extension (ETE) is considered an important prognostic factor in patients with papillary thyroid carcinoma (PTC) [1, 2]. ETE can be classified as minimal or gross, based on the extent of invasion of the tumor to adjacent structures. Both minimal and gross ETE were accepted as important variables during staging of differentiated thyroid carcinoma, regardless of tumor size [3, 4]. However, several studies have reported that minimal ETE has minimal influence on outcomes of the patient [5, 6]. These results were reflected in the eighth edition of TNM staging from the American Joint Committee on Cancer (AJCC), and minimal ETE has been removed from the T stage of differentiated thyroid carcinoma [7].

It is well accepted that patients with gross ETE have a worse outcome compared to those with minimal ETE. However, the oncological significance of minimal ETE still remains controversial. The association of ETE with tumor size and multifocality has been described in previous studies [8–11]. Tumor size is one of the most important prognostic factors in PTC, along with patient age, gender, and metastasis. Although not included in any of the prognostic classification systems, multifocality is generally regarded as a high-risk factor. Accordingly, tumor size and multifocality could be considered confounding variables, when investigating the clinical implications of ETE.

The relationship between ETE and lymph node (LN) metastasis has also been discussed in earlier studies [8, 12, 13], along with BRAF mutation, location of tumor, and iodine intake [14–18]. Controversy still exists on whether LN metastasis affects overall survival [19–21]. However, the association between LN metastasis and locoregional recurrence has been consistently confirmed through previous reports. Patients with recurred disease often require additional operations or radioactive iodine ablation, which can subsequently result in secondary complications.

The aim of this study was to investigate the clinicopathologic characteristics of minimal ETE in patients with single papillary thyroid microcarcinoma (PTMC) and its effects on LN metastasis.

## Methods

### Patients

Medical records of 1994 patients who underwent thyroidectomy for PTC at Chung-Ang University Hospital between 2012 and 2016 were retrospectively reviewed. After exclusion of patients with multifocal tumors and those with tumors larger than 1 cm, 814 patients with single PTMC were included in the study. Patients were according to the presence and extent of ETE into three groups (no ETE vs. minimal ETE vs. gross ETE), as appearing in the permanent pathology reports. Minimal ETE was defined as tumor invasion beyond the thyroid capsule detected on microscopy, and gross ETE was defined as obvious extension of the primary tumor to adjacent structures detected during operation. Gender, age, histologic variation, lymphovascular invasion, lymph node metastasis, BRAF mutation, and extent of surgery were reviewed. The study protocol was approved by the Institutional Review Board of Chung-Ang University Hospital (2203–019-19,411).

### Surgery

Five hundred sixty-one patients received a total thyroidectomy, 244 underwent unilateral lobectomy, and 9 patients had a wide isthmusectomy. Prophylactic central node dissection (CND) was routinely performed on the ipsilateral side of thyroid tumor. Bilateral therapeutic CND was performed on patients who underwent total thyroidectomy when there were suspicious lymph nodes on preoperative imaging studies. Lateral neck dissection was performed in 38 patients, who were preoperatively diagnosed with metastatic lymph nodes.

### Statistical analysis

SPSS version 22 (SPSS, Inc., Chicago, IL, USA) was used for all statistical analyses. Continuous data were compared using analysis of variance (ANOVA). Nominal data were analyzed with chi-square test or Fisher’s exact test. Data are expressed as mean ± standard deviation or number (%). Multivariate analysis was performed using logistic regression to assess the impact of clinicopathological features in association to lymph node metastasis. Results are presented as the odds ratio (OR) with a 95% confidence interval (CI). A *p*-value < 0.05 defined statistical significance.

## Results

### Patients and tumor characteristics according to extent of ETE

Five hundred ninety-three (72.9%) patients had no ETE, 204 (25.1%) had minimal ETE, and 17 (2.1%) had gross ETE (Table 1). There was no significant difference in gender, age, and histologic variation between the groups. Both minimal and gross ETE were significantly associated with lymphatic invasion, perineural invasion, vascular invasion, and LN metastasis compared to the no ETE group. Furthermore, gross ETE showed a higher rate of lymphatic invasion, perineural invasion, vascular invasion, and LN metastasis in contrast to minimal ETE. The proportion of BRAF mutant tumors were significantly higher in the gross ETE group, when compared to the no ETE group (*p* = 0.005) but not the minimal ETE group (*p* = 0.095).

**Table 1:** Clinicopathological features of patients according to the presence and extent of ETE

	No ETE (*n* = 593)	Minimal ETE (*n* = 204)	Gross ETE (*n* = 17)	*p*-value
Gender				0.814
Female	466 (78.6%)	164 (80.4%)	14 (82.4%)	
Male	127 (21.4%)	40 (19.6%)	3 (17.6%)	
Age (years, mean ± SD)	45.8 ± 11.7	46.0 ± 12.1	42.4 ± 14.0	0.482
Histologic variation				0.206
Conventional	385 (64.9%)	154 (75.5%)	12 (70.6%)	
Follicular variant	194 (32.7%)	46 (22.5%)	5 (29.4%)	
Tall cell variant	2 (0.3%)	1 (0.8%)	0 (0%)	
Other	12 (2.0%)	3 (1.5%)	0 (0%)	
Lymphatic invasion	8 (1.3%)	10 (4.9%)	4 (23.5%)	< 0.001
Perineural invasion	4 (0.7%)	7 (3.4%)	2 (11.8%)	< 0.001
Vascular invasion	1 (0.2%)	2 (1.0%)	2 (11.8%)	< 0.001
LN metastasis	187 (31.5%)	96 (47.1%)	11 (64.7%)	< 0.001
Lateral LN metastasis	24 (4.0%)	12 (5.9%)	2 (11.8%)	< 0.001
BRAF mutation	117/191 (61.3%)	135/204 (66.2%)	33/39 (84.6%)	0.309 (a) 0.005 (b) 0.095 (c)
Extent of surgery				< 0.001
Total thyroidectomy	390 (65.8%)	156 (76.5%)	15 (88.2%)	
Lobectomy	201 (33.9%)	41 (20.1%)	2 (11.8%)	
Wide isthmusectomy	2 (0.3%)	7 (3.4%)	0 (0%)	

*ETE* Extrathyroidal extension, *LN* Lymph node

(a) no ETE vs. minimal ETE, (b) no ETE vs. gross ETE, (c) minimal ETE vs. gross ETE

### Clinicopathological features according to LN metastasis

Table 2 shows the clinicopathological differences of patients in relation to lymph node metastasis. LN metastasis was significantly more common in male patients and younger patients. The proportion of patients with ETE was significantly higher in patients with LN metastasis (*p* < 0.001). BRAF mutation was similar between both groups.

**Table 2:** Clinicopathological features of patients according to lymph node metastasis

	No LNM (*n* = 520)	LNM (*n* = 294)	*p*-value
Gender			< 0.001
Female	435 (83.7%)	209 (71.1%)	
Male	85 (16.3%)	85 (28.9%)	
Age (years, mean ± SD)	48.11 ± 11.6	41.7 ± 11.3	< 0.001
Histologic variation			< 0.001
Conventional	315 (60.6%)	236 (80.3%)	
Follicular variant	192 (36.9%)	53 (18.0%)	
Tall cell variant	3 (0.6%)	0 (0%)	
Other	10 (1.9%)	5 (1.7%)	
Lymphatic invasion	5 (1.0%)	17 (5.8%)	< 0.001
Perineural invasion	3 (0.6%)	10 (3.4%)	0.003
Vascular invasion	1 (0.2%)	4 (1.4%)	0.060
ETE			< 0.001
No	406 (78.1%)	187 (63.6%)	
Minimal	108 (20.8%)	96 (32.7%)	
Gross	6 (1.2%)	11 (3.7%)	
BRAF mutation	105/168 (62.5%)	66/98 (67.3%)	0.507
Extent of surgery			< 0.001
Total thyroidectomy	313 (60.2%)	248 (84.4%)	
Lobectomy	201 (38.7%)	43 (14.6%)	
Wide isthmusectomy	6 (1.2%)	3 (1.0%)	

*LNM* lymph node metastasis

### Risk factors for LN metastasis

Clinical and pathological variables that were significant in univariate analysis were included in the multivariate analysis regression for assessment as risk factors of lymph node metastasis (Table 3). Male gender, younger age, lymphatic invasion, perineural invasion, and minimal ETE were independently associated with LN metastasis. Patients with gross ETE were 2.1 times more likely to be accompanied by LN metastasis (95% CI 0.656–6.997) but with no statistical significance (*p* = 0.207).

**Table 3:** Multivariate logistic regression of lymph node metastasis in single PTMC

	Adjusted OR	95% CI	*p*-*v*alue
Male	1.987	1.369–2.884	< 0.001
Age (years, mean ± SD)	0.953	0.939–0.967	< 0.001
Histologic variation			
Conventional			Reference
Follicular variant	0.473	0.326–0.686	< 0.001
Tall cell variant			0.999
Other			0.792
Lymphatic invasion	4.389	1.522–12.658	0.006
Perineural invasion	6.545	1.262–33.948	0.025
Vascular invasion	4.564	0.460–45.266	0.195
ETE			
No			Reference
Minimal	1.852	1.298–2.643	0.001
Gross	2.143	0.656–6.997	0.207

*PTMC* Papillary thyroid microcarcinoma, *OR* Odds ratio, *CI* Confidence interval, *ETE* Extrathyroidal invasion

### Recurrence

The mean follow-up period was 30.0 ± 15.7 months (range, 1 ~ 61 months). Recurrence was identified in 3 (0.4%) patients during follow-up. All three patients recurred in the lateral neck LN, and there were no distant metastases.

## Discussion

Minimal ETE was first implemented in the TNM criteria by the AJCC in 2002. Differentiated thyroid carcinoma with minimal ETE was classified as T3, which upstaged tumors smaller than 4 cm [3]. Recent changes in the eight edition of the AJCC TNM staging system reflect the results from various studies that have invalidated the effects of minimal ETE on overall survival and mortality. Nonetheless, the oncological significance of minimal ETE still remains controversial. Minimal ETE is regarded in association with tumor size, multifocality, LN metastasis, and recurrence [8, 10–12, 19, 22, 23].

Papillary thyroid microcarcinoma is defined as a papillary thyroid carcinoma ≤ 1 cm in maximum diameter. PTMC is commonly viewed as non-progressive, due to its relatively indolent course. Disease-free survival and overall survival rate are commonly discussed when estimating the prognosis of a particular disease. Hay et al. reported cause-specific mortality rates of 0.1, 0.1, and 0.7% at 10, 20, and 40 years, respectively, in a study of 900 cases of PTMC during a 60-year period [24]. Many studies have shown that LN metastasis in PTMC is closely related to recurrence and disease-free survival [25, 26]. A retrospective study by Karatzas et al. concluded that LN metastasis was the strongest prognostic factor of tumor recurrence in patients with nonincidental PTMC [27].

In this study, we investigated the clinical and pathologic characteristics of ETE in 814 patients with single PTMC by excluding the effects of tumor size and multifocality. No ETE and gross ETE were also included for analysis in order to see the differences in this specific subset of patients with single PTMC, according to the extent of ETE, with emphasis on minimal ETE. Furthermore, the risk factors for LN metastasis in single PTMC were also assessed. Few studies have assessed the value of minimal ETE in PTMC. Moon et al. included 288 patients with conventional PTMC and found that minimal ETE was associated with tumor size and central LN metastasis but not recurrence [28]. Woo et al. evaluated the significance of minimal ETE in 144 patients with solitary PTMC and concluded that it had no significant influence on recurrence-free survival. This study included patients with 814 patients with single PTMC, which is a relatively larger population, compared with the former studies.

Minimal ETE was significantly associated with lymphatic invasion, perineural invasion, vascular invasion, and LN metastasis compared to the no ETE group but less than the gross ETE group. Minimal ETE was independently associated with LN metastasis, along with male gender, and younger age. These results are consistent with previous studies [8, 10, 12]. Since there were no mortality and only three cases of recurrence during 30 months (range, 1 ~ 61 months) of follow-up, we could not assess survival outcomes.

Two recent systematic reviews and meta-analyses that studied the impact of minimal ETE in differentiated thyroid cancer concluded that minimal ETE increases the risk of recurrence in PTC [29, 30]. In patients with PTMC, the impact of minimal ETE was non-significant [30]. We agree with previous reports that minimal ETE has no impact on survival, and the increase in risk of recurrence is small. However, LN metastasis in PTMC has been associated with recurrence. Thus, when there is suspicion of ETE at the time of operation, examination of the cervical lymph nodes is suggested. Similarly, in situations where ETE is suspected during active surveillance of small PTCs, careful inspection of metastatic LNs may be required.

A recent study of PTMC patients in Cyprus concluded that the prevalence of LN metastasis and ETE was especially higher in patients with multifocal tumors [31]. The main purpose of our study was to evaluate the effects of minimal ETE, and the associated LN metastasis in PTMC patients, which is why we chose to only include those with single tumors. However, multifocality is also associated LN metastasis and should be carefully looked for while assessing patients with PTMC.

There are some limitations to this study. The relatively short follow-up period and low recurrence rate made it difficult to assess the long term effects of minimal ETE in single PTMC patients. Most of the patients in the study were operated before the updates in thyroid cancer management guidelines were published in 2015 [32], and all patients received prophylactic central neck dissection, which may have impacted the number of LN metastasis detected. One may argue that information gained from prophylactic node dissection may upstage patients, leading to overtreatment. However, this information cannot be devalued, especially while managing PTMC patients before and after surgery.

## Conclusions

Minimal ETE was significantly associated with lymphatic invasion, perineural invasion, vascular invasion, and LN metastasis in patients with single PTMC. Minimal ETE was independently associated with LN metastasis. Minimal ETE should be considered during the decision making in managing of patients with single PTMC, whether it be surgical or for active surveillance.

## Data Availability

The datasets used and/or analyzed during the current study are available from the corresponding author on reasonable request.

## Abbreviations

ETE: Extrathyroidal extension
PTC: Papillary thyroid carcinoma
PTMC: Papillary thyroid microcarcinoma
LN: Lymph node
CND: Central node dissection
OR: Odds ratio
CI: Confidence interval
